# A novel BMI-1 inhibitor QW24 for the treatment of stem-like colorectal cancer

**DOI:** 10.1186/s13046-019-1392-8

**Published:** 2019-10-22

**Authors:** Jinhua Wang, Yajing Xing, Yingying Wang, Yundong He, Liting Wang, Shihong Peng, Lianfang Yang, Jiuqing Xie, Xiaotao Li, Wenwei Qiu, Zhengfang Yi, Mingyao Liu

**Affiliations:** 1 East China Normal University and Shanghai Fengxian District Central Hospital Joint Center for Translational Medicine, Shanghai Key Laboratory of Regulatory Biology, Institute of Biomedical Sciences and School of Life Sciences, East China Normal University, Shanghai, 200241 China; 2 Shanghai Engineering Research Center of Molecular Therapeutics and New Drug Development, School of Chemistry and Molecular Engineering, East China Normal University, Shanghai, 200062 China; 3 Department of Molecular and Cellular Biology, Dan L. Duncan Cancer Center, Baylor College of Medicine, Houston, TX 77030 USA

**Keywords:** Colorectal cancer, Cancer stem-like, BMI-1

## Abstract

**Background:**

Cancer-initiating cell (CIC), a functionally homogeneous stem-like cell population, is resonsible for driving the tumor maintenance and metastasis, and is a source of chemotherapy and radiation-therapy resistance within tumors. Targeting CICs self-renewal has been proposed as a therapeutic goal and an effective approach to control tumor growth. BMI-1, a critical regulator of self-renewal in the maintenance of CICs, is identified as a potential target for colorectal cancer therapy.

**Methods:**

Colorectal cancer stem-like cell lines HCT116 and HT29 were used for screening more than 500 synthetic compounds by sulforhodamine B (SRB) cell proliferation assay. The candidate compound was studied in vitro by SRB cell proliferation assay, western blotting, cell colony formation assay, quantitative real-time PCR, flow cytometry analysis, and transwell migration assay. Sphere formation assay and limiting dilution analysis (LDA) were performed for measuring the effect of compound on stemness properties. In vivo subcutaneous tumor growth xenograft model and liver metastasis model were performed to test the efficacy of the compound treatment. Student’s t test was applied for statistical analysis.

**Results:**

We report the development and characterization of a small molecule inhibitor QW24 against BMI-1. QW24 potently down-regulates BMI-1 protein level through autophagy-lysosome degradation pathway without affecting the BMI-1 mRNA level. Moreover, QW24 significantly inhibits the self-renewal of colorectal CICs in stem-like colorectal cancer cell lines, resulting in the abrogation of their proliferation and metastasis. Notably, QW24 significantly suppresses the colorectal tumor growth without obvious toxicity in the subcutaneous xenograft model, as well as decreases the tumor metastasis and increases mice survival in the liver metastasis model. Moreover, QW24 exerts a better efficiency than the previously reported BMI-1 inhibitor PTC-209.

**Conclusions:**

Our preclinical data show that QW24 exerts potent anti-tumor activity by down-regulating BMI-1 and abrogating colorectal CICs self-renewal without obvious toxicity in vivo, suggesting that QW24 could potentially be used as an effective therapeutic agent for clinical colorectal cancer treatment.

**Electronic supplementary material:**

The online version of this article (10.1186/s13046-019-1392-8) contains supplementary material, which is available to authorized users.

## Background

Colorectal cancer (CRC) is the third most commonly diagnosed cancer among both men and women in the United States [1]. CRC is also the second leading cause of cancer death in men and the third in women in the United States [2]. In 2018, approximately 1.1 million individuals developed colorectal cancer globally and the disease-specific mortality rate is about 50% [3]. Treatment for patients with cancers of colon and rectum contains surgical resection, chemotherapy and radiation therapy [4]. However, cancer recurrence is still common among colorectal survivors; approximately one-half of patients treated with surgery will experience a recurrence within the first 3 years after surgery [5]. Colorectal cancer survivors are also at increased risk of second primary cancers of the colon and rectum, as well as other cancer sites, especially those within the digestive system [5]. Evidences suggest that a subpopulation of cancer cells, called cancer stem cells (CSCs) or cancer-initiating cells (CICs), has a central role in colorectal cancer recurrence [6, 7]. CICs initiate and sustain tumor growth because of the biological characteristics including resistance to treatment [8], evasion of cell death, self-renewal and dormancy [6]. After CRC diagnosis, the primary tumor is usually removed by curative or palliative surgery, which may follow a neoadjuvant chemotherapy [9]. In case of CICs dissemination, chemotherapy may kill a significant proportion of CICs, which are not protected by the tumor microenvironment. However, some residual CICs that survive chemotherapy may produce liver metastases that, in turn, need to be treated with effective therapies [10]. While chemotherapy is present in all the current therapeutic regimens for CRC, additional therapeutics targeting major CIC pathways (anti-CICs) are required to achieve a potent cure in advanced disease, particularly in the absence of specific therapies targeting driver oncogenes [11]. Numerous researches have proved that targeting CIC self-renewal could be an efficient way to control tumor growth and metastasis [12–14].

BMI-1 is the key regulatory component of polycomb repressive complex 1 (PRC1) that influences chromatin structure and regulates transcriptional activity of many genes, such as the tumor suppressor p16 and p14 [15]. BMI-1 represents the most important regulator that closely relates to the self-renewal of CICs and maintains the stemness of CICs [16]. Overexpression of BMI-1 is closely related with tumor progression by the involvement in tumor initiation, metastasis, invasion and chemoresistance within various cancer types [17]. The high expression of BMI-1 is correlated with poor prognosis so that BMI-1 is considered as a viable therapeutic target in some malignancies, such as colorectal cancer [18, 19], ovarian cancer [20, 21], prostate cancer [22, 23], non-small lung cancer [24], breast cancer [25, 26]. A number of microRNAs and small-molecule compounds targeting BMI-1 regulating stemness have been identified [18, 20, 23, 27–29]. It has been reported that a small molecule compound PTC-209, the inhibitor of BMI-1, irreversibly impair colorectal CICs by impacting self-renewal [18]. Therefore, targeting the BMI-1-related self-renewal machinery provides the basis for a new therapeutic approach in the treatment of colorectal cancer.

We describe here that QW24, a novel synthetic small molecule compound, impairs the self-renewal of CICs and inhibits the growth and metastasis of colorectal cancer in xenograft models by down-regulating BMI-1 significantly through autophagy-lysosome protein degradation pathway. Thus, QW24 could be a potential and potent drug candidate for colorectal cancer therapy.

## Materials and methods

### Cells culture and animals

Human colorectal cancer cell lines HCT116, HT29, HCT8, HCT15, LS174T, LoVo, mouse colon cancer cell line CT26 and normal cell lines NCM460, L02 and HAF were obtained from the American Type Culture Collection (ATCC). HUVEC were obtained from ScienCell Research Laboratories. HCT116, HT29, HCT8, HCT15, LoVo and CT26 were cultured in RPMI-1640, LS174T was maintained in DMEM, and HUVEC was cultured with ECM. All the media were obtained from Gibco. Medium was supplemented with 10% FBS, penicillin (100 units/ml) and streptomycin (100 units/ml). BALB/c nude mice and BALB/c mice were from the National Rodent Laboratory Animal Resources, Shanghai Branch of China. All animal experimental protocols were approved by the Animal Investigation Committee of the Institute of Biomedical Sciences, East China Normal University.

### Sulforhodamine B (SRB) cell proliferation assay

SRB cell proliferation assay were performed as previously described [30]. Cells were seeded in 96-well plates (3000 cells/well) and incubated with indicated concentrations of QW24 or other small molecular compounds. After incubation for indicated time, the cells were then fixed with trichloroacetic acid, stained with SRB (Sigma Aldrich, Argentina, Cat# S1402), and analyzed for percent of survival on 96-well plate reader. The IC50 (half maximal inhibitory concentration) value was calculated using GraphPad software.

### Western blotting and antibodies

Cells were lysed in cell lysis buffer (50 mM Tris-HCl, pH 7.5, 150 mM NaCl, 1% Nonidet P-40, 0.5% sodium deoxycholate and 1% protease inhibitor cocktails) and boiled for 10 min with loading buffer (2% SDS, 10% glycerol, 10% β-Mercaptoethanol, Bromphenol Blue and Tris-HCl, pH 6.8). Lysates were fractionated on polyacrylamide gels and transferred to nitrocellulose. The blots were probed with specific antibodies followed by secondary antibody, then membranes were examined by the LI-COR Odyssey infrared imaging system (LI-COR Biotechnology, Lincoln NE). BMI-1 antibody (Catalog #ab14389) was from Abcam, H2A antibody (Catalog #12349) and Ub-H2A antibody (Catalog #8240) were from Cell Signaling Technology. β-actin antibody (Catalog #A5441) and LC3 antibody were from Sigma. The secondary antibody was conjugated with IRDye 680/800 (Catalog #926–32,221, #926–32,210, Millennium Science).

### Cell colony formation assay

Cell colony formation assay was performed as described previously [30, 31]. Colorectal cancer cells HCT116, HT29 and HCT8 were seeded 2000 per well in 6-well plate and allowed to grow for 24 h. Then cells were treated with indicated concentrations of PTC-209 or QW24 for 7 days. Colonies were fixed with 4% paraformaldehyde for 30 min, stained with 0.1% crystal violet for 5 min. Colonies were visualized under a microscope, and all the fields were imaged and counted. Colony formation as a percentage of vehicle control for each cell line is presented.

### Sphere formation assay

Sphere formation assay was performed as reported previously [18, 32]. HCT116 cells were cultured in 96-well clear flat bottom ultra-low attachment microplate (catalog #3474, Corning) with serum-free medium (SFM) (DMEM/F12) supplemented with 20 ng/ml of basic fibroblast growth factor (FGF) and epidermal growth factor (EGF) (PeproTech), 5 μg/ml of insulin (Sigma–Aldrich), 0.4% bovine serum albumin (BSA, Invitrogen), and 2% B27 (Invitrogen). Culture medium was replaced or supplemented with additional growth factors twice a week.

### Limiting dilution analysis (LDA)

For in vitro LDAs, QW24 pretreated HCT116 cells were dissociated into single cells and seeded in 96-well plates at indicated cell doses. For each cell dose, at least 8 wells were seeded with cells, and for the lower cell doses (10 cells or single cell per well), 12 wells were plated. After 1 week incubation, wells containing spheres were scored, and the number of positive wells was used to calculate the frequency of sphere-forming units using the ELDA software (http://bioinf.wehi.edu.au/software/elda/index.html) provided by the Walter and Eliza Hall Institute [33].

### Quantitative real-time PCR

Cells were treated with the indicated concentration of QW24 for indicated time, and total RNA was extracted using TRIzol (Takara, Japan) according to the manufacturer’s instructions. 1 μg of total RNA was used for cDNA synthesis using a cDNA reverse transcription kit (Takara, Japan). Real-time PCR was performed in triplicates using gene-specific primers on a Stratagene Mx3005P PCR system (Agilent Technologies) machine. The mRNA expression levels were normalized to β-actin. The specific primer for BMI-1 and β-actin are BMI-1-F: TGGAGAACTGGAAAGTGACTCTGG; BMI-1-R: AAGAAGATTGGTGGTTACCGCTG; β-actin-F: TGAAGTGTGACGTGGACATC; β-actin-R: CATACTCCTGCTTGCTGATC. All analysis was performed using the Microsoft Excel and GraphPad Prism 6 software.

### Flow cytometry analysis

After treatment with different concentrations of QW24 or PTC-209, cells were trypsinized, washed with PBS and stained with CD44-PE (Catalog #561861, BD Biosciences) antibody and CD133-APC (Catalog #130–113–668, Miltenyi Biotec) antibody for 15 min at 4 °C in staining buffer (PBS + 0.5% BSA) in the dark. The stained cells were analyzed using BD LSRII flow cytometry (BD Biosciences).

### Transwell migration assay

Transwell migration assay was performed as described previously [34]. Cell migration assays were performed using a 24-well chamber containing a membrane of 8 μm pore size (Millipore). HCT116, HT29 and CT26 cells were firstly cultured in serum-free medium for 12 h. Then, 100 μl cell suspension with 4 × 10^4^ cells were added into the upper chamber, and the bottom chamber was filled with 600 μl culture medium containing 10% FBS. After incubation for 12 h at 37 °C, the cells were fixed with 4% paraformaldehyde and stained using 0.1% crystal violet. The non-invaded cells on the upper membrane surface were removed with a cotton tip. Three replicates were performed for each group and the numbers of invaded cells were counted in 6 randomly selected high-power fields under a microscope (Leica, Heidelberg, Germany).

### Lentiviral packing and BMI-1 overexpression

BMI-1 construct was cloned into the lentiviral vector pTSiN, and virus production was performed as described previously [35]. 293 T cells were co-transfected with pTSiN empty vector or pTSiN-BMI-1 vector along with packing and envelop plasmids by Lipofectamine 2000 according to the manufacturer’s instructions. At 2 days post-transfection, HCT116 cells were transduced by culturing with a 1:0.5 or 1:2 mixture of fresh medium and virus supernatant with polybrene (4 μg/ml final concentration) for 72 h. Then the infected cells were treated with indicated concentration of PTC-209 or QW24.

### In vivo subcutaneous tumor growth xenograft models

As reported previously [36, 37], HCT116 cells (3 × 10^6^ cells/mouse) were subcutaneously injected into male BALB/c-nude mice (6–8 weeks of age). After the volume of tumor nodules reached approximately 100 mm^3^, the mice were randomly assigned to the indicated groups and the mice were injected intraperitoneally (i.p.) with DMSO vehicle (*n* = 10), PTC-209 30 mg/kg (*n* = 10), QW24 15 mg/kg (*n* = 10), QW24 30 mg/kg (*n* = 10) for 24 days, with the measurement of bodyweight and the tumor dimensions. The tumor volume was calculated using the following equation: tumor volume = length × width × width × 0.52 [38].

### Liver metastasis model

For colorectal cancer liver metastasis study, male BALB/c mice (6–8 weeks of age) were anesthetized using 150 mg/kg 2, 2, 2-tribromethanol plus 350 mg/kg tert-amyl alcohol and then 1 × 10^6^ CT26-luciferase colon cancer cells were surgically injected into the spleens as described previously [39]. One week after injection, mice were administrated with splenectomy and mice were randomized into three groups. Then mice were intraperitoneally (i.p) injected with PTC-209 30 mg/kg (*n* = 9), QW24 30 mg/kg (*n* = 10) every day for 12 days, with an equivalent volume of dimethyl sulfoxide (DMSO) was injected in vehicle group (*n* = 9). Hepatic metastases were monitored every 3 days using the IVIS Imaging System (Xenogen Corporation, Alameda, CA). Images and measurements of bioluminescent signals were acquired and analyzed using Living Image and Xenogen software [30]. For survival study, we used the same animal model as descripted previously in this section. The mice were treated with DMSO (*n* = 10), PTC-209 30 mg/kg (*n* = 10) and QW24 30 mg/kg (*n* = 10) every day for 21 days, and the survival of the mice were recorded to 14 weeks and Kaplan-Meier survival curve was analyzed.

### Histology and immunohistochemistry (IHC)

Tumors or mice tissue specimens were excised after sacrificing mice and specimens were immediately fixed in 10% neutral buffered formaldehyde for 24 h, progressively dehydrated in solutions containing an increasing percentage of ethanol (75, 85, 95 and 100%, v/v), and embedded into paraffin blocks. Sections were cut from the paraffin blocks and IHC was carried out using anti–Ki-67 (1:250; Catalog #ab15580, Abcam), anti-PCNA (1:250; Catalog #ab18197, Abcam) and anti-BMI-1 (1250; Catalog #ab14389, Abcam) as primary antibodies. For hematoxylin-eosin (H&E) staining, samples were stained with H&E to indicate nucleus and cytoplasm, respectively.

### Statistical analysis

The differences between control group and experimental groups were determined by Student’s t test. Data was expressed as mean and standard deviation (s.d.) and *P* < 0.05 was considered significant.

## Results

### QW24 inhibits colorectal cancer cell growth and down-regulates BMI-1

In the process of cancer development and progression, the basic characteristic of cancer is that the tumor cells proliferate immortally [6]. Inhibiting cancer cells proliferation would be the primary standard for small molecule compounds screening. To this end, we used colorectal cancer stem-like cell lines HCT116 and HT29 [40, 41] for screening more than 500 synthetic compounds (Additional file 1: Figure S1A). HCT116 cell line was identified as cancer stem-like cells with the high expression of CSCs markers CD133 and CD44 [42, 43]. Moreover, HCT116 was identified as stem-like by immunostaining analyses for the differentiation marker KRT20 [41]. Furthermore, it was characterized that the HCT116 cell line does not express CDX1, which regulates intestine-specific gene expression and enterocyte differentiation [44]. Thus, HCT116 has no ability of differentiation and contains mainly CSCs [44]. As colorectal cancer stem-like cell, HCT116 was widely used in various studies [45–48]. HT29 cell line was identified as cancer stem-like cells with the high expression of CSCs markers CD44 and CD24. CD44^+^ CD24^+^ cells are enriched in the HT29 cell line, which is clonogenic in vitro and can initiate tumors in vivo [44]. As colorectal cancer stem-like cell, HT29 was widely used in various studies [49–52]. Both HCT116 and HT29 are spheroid-derived cells that have high self-renewal capacity [53].

**Fig. 1:**
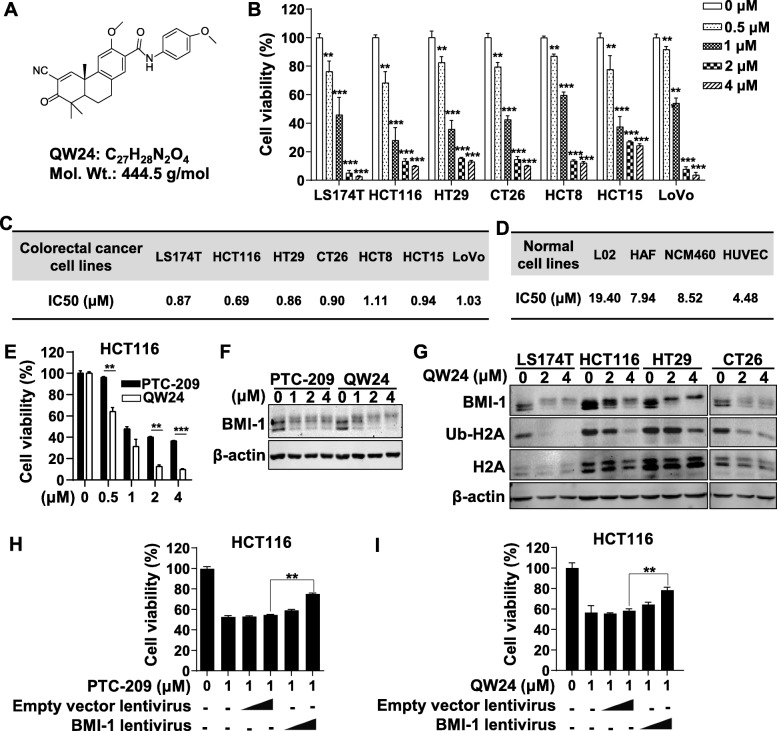
QW24 inhibited colorectal cancer cells proliferation and down-regulated BMI-1. **a**, Chemical structure of QW24 with the molecular weight (Mol. Wt.) of 444.5 g/mol. **b**, LS174T, HCT116, HT29, CT26, HCT8, HCT15 and LoVo cells were seeded in 96-well plates (3000 cells/well) and treated with 0, 0.5, 1, 2, 4 μM of QW24 after cells were attached. After 72 h incubation, cell growth was determined by SRB assay. **c**, The IC50 of QW24 for different colorectal cancer cell lines were listed. **d**, The IC50 of QW24 for normal cell lines were listed, including human normal liver cell L02, human skin fibroblast cell HAF, human normal colon epithelium cell NCM460 and human umbilical vein endothelial cell HUVEC. **e**, HCT116 cells were seeded in 96-well plates (3000 cells/well) and treated with 0, 0.5, 1, 2, 4 μM of QW24 or PTC-209 after cells were attached. After 72 h incubation, cell growth was determined by SRB assay. **f**, HCT116 cells were treated with 0, 1, 2, 4 μM of QW24 or PTC-209 for 12 h. Cells were lysed and BMI-1 protein level was measured by western blotting analysis. **g**, LS174T, HCT116, HT29, and CT26 cells were treated with 0, 2, 4 μM of QW24 for 12 h. Cells were lysed and BMI-1, Ub-H2A and H2A protein levels were measured by western blotting analysis. **h** and **i**, HCT116 cells were infected with increasing doses of empty vector lentivirus (control) or BMI-1 lentivirus for 72 h. Cells were seeded in 96-well plates (3000 cells/well) and treated with indicated concentrations of PTC-209 (**h**) or QW24 (**i**) after cells were attached. After 96 h incubation, cell growth was measured by SRB assay. Data are presented as mean ± s.d. (*n* = 5); **, *P* < 0.01; ***, *P* < 0.001

We detected the influence of numerous compounds to colorectal cancer cells proliferation of HCT116 and HT29 by SRB assay and determined the IC50 (half maximal inhibitory concentration) of the compounds (Additional file 1: Figure S1A). The previously reported BMI-1 inhibitor PTC-209 [18] has the IC50 of 1.67 μM in HCT116 cells and 1.36 μM in HT29 cells based on our results (Additional file 1: Figure S1B). Thus, we selected the four compounds QW07, QW30, QW10 and QW24 (chemical structure shown in Additional file 1: Figure S1C and Fig. 1a) as candidates whose IC50s were less than 1 μM in HCT116 and HT29 cells (Additional file 1: Figure S1B), suggesting they had potent activity comparable to PTC-209. As reported [18], BMI-1, the primary regulator of colorectal CICs, could be the critical target for colorectal cancer therapy. Therefore, cells were treated with the candidate compounds for 12 h respectively and then we detected their effects on the protein level of BMI-1 by western blotting. Finally, we achieved the only compound QW24 that dramatically suppressed cancer cells proliferation and down-regulated BMI-1 simultaneously. By contrast, the other three compounds have no obvious effect on BMI-1 protein level (Additional file 1: Figure S1D). To verify the results further, we increased the treatment concentrations of compounds and extended the treatment time to 24 h. The results showed that QW24 decreased the protein level of BMI-1 dramatically, however, QW30 with lower IC50 had no effect even if the treatment concentration was increased to 10 μM (Additional file 1: Figure S1E). These results together confirm that QW24 potently inhibits the colorectal cancer stem-like cells proliferation and decreases the protein level of BMI-1 significantly.

To investigate the effect of QW24 on the proliferation of colorectal cancer cells, we used several human colorectal cancer cell lines, including LS174T, HCT116, HT29, HCT8, HCT15, LoVo, and mouse colon cancer cell line CT26, with SRB assay. The results showed that QW24 inhibited the proliferation of colorectal cancer cell lines dramatically with concentration dependency, and the IC50 values for all cell lines were around 1.0 μM (Fig. 1b and c). To explore the toxicity of QW24 on normal cells, the IC50 of QW24 for four normal cell lines was determined, including human normal liver cell L02, human skin fibroblast cell HAF, human normal colon epithelium cell NCM460 and human umbilical vein endothelial cell HUVEC. The IC50 values for normal cells were significant higher than cancer cells (Fig. 1c, Fig. 1d and Additional file 1: Figure S1F), indicating that QW24 has lower toxicity to normal cells. Moreover, to compare the inhibitory effects of QW24 with PTC-209 on cancer stem-like cell growth, the stem-like cells HCT116 were treated with QW24 or PTC-209 with same concentration and time. The results showed that QW24 had comparable anti-cancer activity as PTC-209, and QW24 even had more significant effect of inhibiting cancer cell proliferation than PTC-209 (Fig. 1e). The other colorectal cancer lines were also treated with PTC-209 or QW24 and the results also showed that QW24 inhibited the cell proliferation more significantly than PTC-209 (Additional file 2: Figure S2B), as well as the results of colony formation assay (Additional file 2: Figure S2A). Moreover, QW24 down-regulated BMI-1 expression more obviously than PTC-209 with the treatment concentrations of 2 μM and 4 μΜ (Fig. 1f).

We determined the protein levels of BMI-1 in different cancer cells and normal cells by western blotting (Additional file 3: Figure S3A). The expression levels of BMI-1 were lower in normal cells compared to cancer cells, which was consistent with the result that QW24 has lower toxicity to normal cells. QW24 impacts the colony formation and cell viability of colorectal cancer cells by specifically decreasing BMI-1, while normal cells with minimal BMI-1 expression are much less affected. The specificity of QW24 towards BMI-1 was demonstrated by the fact that normal cells expressing low levels of BMI-1 were nonresponsive while colorectal cancer cells expressing higher BMI-1 showed a dose-dependent decrease in cell viability upon treatment with QW24. Furthermore, the Cancer Genome Atlas (TCGA) database showed BMI-1 was significantly overexpressed in colorectal tumors compared with normal tissues in patients (Additional file 3: Figure S3B), and the cases with higher expression levels of BMI-1 showed worse survival rates than cases with lower expression levels (Additional file 3: Figure S3C).

As mentioned above, BMI-1 is one of the key components in PRC1 complex, which induces the ubiquitination of H2AK119. To further evaluate the effect of QW24 on this pathway, we selected three representative human colorectal cancer cell lines (HCT116, HT29 and LS174T) that were more sensitive (with lower IC50) to QW24 (Fig. 1c). Ub-H2A and H2A level were determined with the treatment of QW24 at 2 μM and 4 μΜ. The result showed that QW24 downregulated BMI-1 and its downstream Ub-H2A, but had no obvious influence on H2A (Fig. 1g**)**. To further confirm the inhibitory effect of QW24 on cell growth through down-regulating BMI-1, HCT116 cells were transduced with lentivirus overexpressing BMI-1 and empty vector lentivirus as control. As shown in Fig. 1h and i, BMI-1 overexpressed cells had reduced sensitivity to the growth inhibitory effects of QW24, with the similar tendency as PTC-209. Taken together, these results indicate that QW24 significantly inhibits colorectal cancer stem-like cell growth by reducing BMI-1 protein level.

### QW24 decreases the stability of BMI-1 protein

**Fig. 2:**
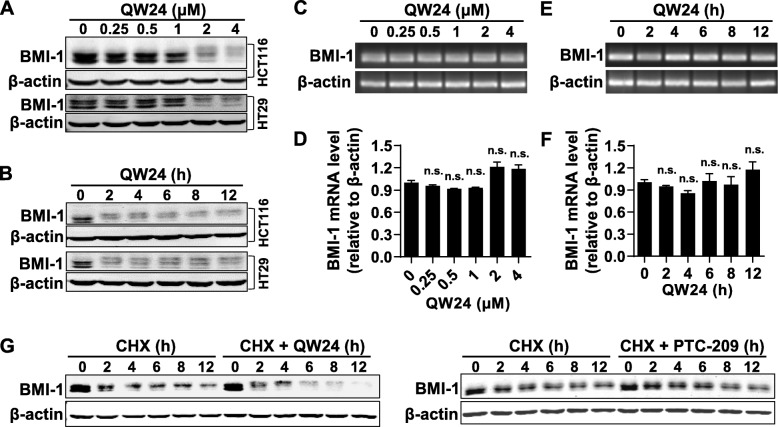
QW24 decreased the protein level of BMI-1 by reducing protein stability. **a** and **b**, HCT116 cells were treated with indicated concentrations of QW24 for 12 h (**a**) or 2 μM QW24 for indicated time (**b**). Cells were lysed and BMI-1 protein level was measured by western blotting analysis. **c-f**. HCT116 cells were treated with indicated concentrations of QW24 for 12 h (**c**, **d**) or 2 μM QW24 for indicated time (**e**, **f**). Then BMI-1 mRNA level was measured by quantitative-PCR. The agarose gel (**c**, **e**) and quantitative-PCR (**d**, **f**) were run to quantify the mRNA level of BMI-1. Data are presented as mean ± s.d. (*n* = 3) in quantitative-PCR; n.s., Not statistically significant. **g**, HCT116 cells were treated with 50 μg/ml protein synthesis inhibitor cycloheximide (CHX) with or without 2 μM QW24 (or 2 μM PTC-209) for indicated lengths of time. BMI-1 protein level was measured by western blotting analysis

QW24 had significant effect on suppressing colorectal cancer stem-like cell growth in vitro by down-regulating BMI-1 protein. However, it was still unclear about how QW24 reduced the protein level of BMI-1. As reported [18], PTC-209 down-regulated BMI-1 by decreasing the transcription of BMI-1. Thus, we firstly determined the effect of QW24 on the transcription of BMI-1. The protein level of BMI-1 was decreased noticeably (Fig. 2a and b), whereas there was no significant change with the mRNA level of BMI-1 at same treatment condition (Fig. 2c-f). These results suggested that QW24 might decrease the stability of BMI-1 protein. Then we treated cells with cycloheximide (CHX), which blocked the protein synthesis in eukaryote cells, to detect the stability of BMI-1 when treated with QW24. As shown in Fig. 2g, compared with CHX treatment alone, the protein level of BMI-1 decreased more significantly with the co-treatment of CHX and QW24, indicating QW24 shortened the lifetime of BMI-1 protein and decreased the protein stability. However, PTC-209 had no obvious effect on the BMI-1 protein stability. Thus, QW24 has different mechanism with PTC-209 for down-regulating BMI-1 that QW24 decreases the stability of BMI-1 protein by inducing protein degradation.

### QW24 degrades BMI-1 protein by autophagy-lysosome pathway

**Fig. 3:**
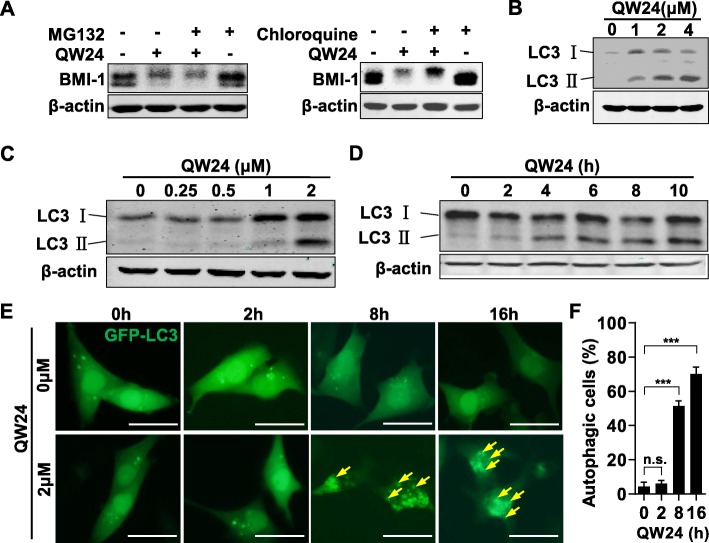
QW24 degraded BMI-1 protein through autophagy-lysosome pathway. **a**, HCT116 cells were pretreated with proteasome inhibitor MG132 (10 μM) or lysosomal inhibitor Chloroquine (100 μM) for 2 h as indicated, and then 2 μM QW24 was added to cells for 6 h. Cells were lysed and BMI-1 protein level was measured by western blotting analysis. **b**, HCT116 cells were treated with indicated concentrations of QW24 for 12 h and LC3 (the autophagy marker) protein level was measured by western blotting analysis. **c**, **d**, HCT116 cells were treated with indicated concentrations of QW24 for 12 h (**c**) or 2 μM QW24 for indicated time (**d**). Then cells were lysed and LC3 protein level was measured by western blotting analysis. **e** and **f**, HCT116 cells were transfected with GFP-LC3 plasmid and treated with 2 μM QW24. Cells were imaged by fluorescence microscope at the indicated time (**e**), and the punctate cytoplasmic LC3 distribution cells were counted (**f**). Scale bars, 10 μm. Data are presented as mean ± s.d. (*n* = 5); n.s., Not statistically significant; ***, *P* < 0.001

The main protein degradation pathways in eukaryote cells are ubiquitin-proteasome pathway and autophagy-lysosome pathway [54]. In ubiquitin-proteasome pathway, cellular proteins that are destined for degradation are marked by the covalent attachment of multiple ubiquitin molecules, which provide a recognition signal for the proteasome [55]. In autophagy-lysosome pathway, targeted proteins are sequestered by phagophores that mature into autophagosome, and then delivered into lysosomes for degradation by autophagosome-lysosome fusion. Chloroquine, the lysosomal inhibitor, blocks autophagy mainly by impairing autophagosome fusion with lysosomes [56]. Firstly, the protease inhibitor MG132 [55] was used to detect whether BMI-1 protein was degraded by ubiquitin-proteasome pathway. The results showed that MG132 treatment did not prevent the decreasing of BMI-1 protein level, indicating the decreasing effect was not rescued (Fig. 3a). However, the treatment with lysosome inhibitor Chloroquine significantly rescued QW24 induced down-regulation of BMI-1 protein level (Fig. 3a). Therefore, we concluded that QW24 degraded BMI-1 protein by the autophagy-lysosome pathway. To validate this conclusion further, the autophagy maker LC3 was detected. The western blotting results showed that the level of LC3 II increased in dose-dependent and time-dependent manners with QW24 treatment (Fig. 3b-d), indicating QW24 induced the down-regulation of BMI-1 through autophagy-lysosome pathway. In addition, we transfected GFP-LC3 plasmid to HCT116 cells and there were autophagosome formed with QW24 treatment (Fig. 3e and f), which provided more evidence for the activation of autophagy-lysosome pathway. Overall, these results indicated that QW24 induced BMI-1 protein degradation through autophagy-lysosome pathway.

### QW24 decreases the self-renewal of colorectal CICs and migration of colorectal cancer cells

**Fig. 4:**
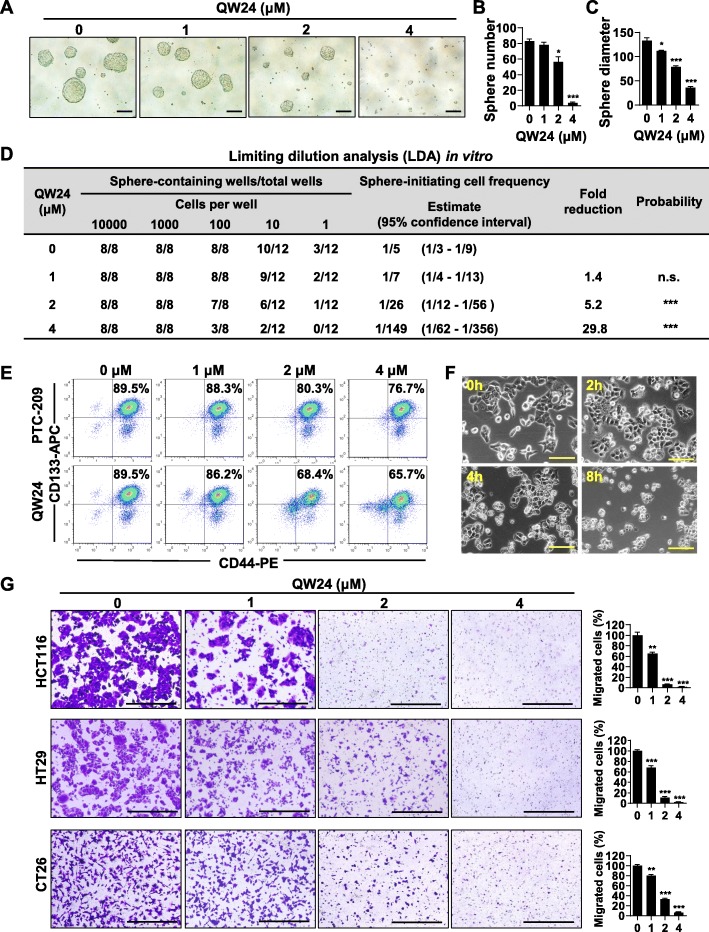
QW24 decreases the self-renewal of colorectal CICs and migration of colorectal cancer cells. **a-c**, HCT116 cells were pretreated with indicated concentrations of QW24 for 4 h. The pretreated cells were cultured in unattached 96-well plates (1000 cells/well) to form the sphere as mentioned in the **Methods**, and the spheres images were taken after 1 week (**a**). The sphere number (**b**) and diameter (**c**) were measured. Scale bars, 100 μm. Data are presented as mean ± s.d. (*n* ≥ 8); *, *P* < 0.05; ***, *P* < 0.001. **d**, QW24 pretreated HCT116 cells were seeded in unattached 96-well plates (1000 cells/well) and cultured for 1 week. The percentage of wells with spheres was determined and computed with limiting dilution analysis (LDA) to determine the sphere-initiating cell frequency. Frequency and probability estimates were computed using the ELDA software. n.s., Not statistically significant; ***, *P* < 0.001. **e**, HCT116 cells were treated with the indicated concentrations of PTC-209 or QW24 for 12 h, and cells were trypsinized, washed with PBS and stained with CD44-PE and CD133-APC antibodies. The stained cells were analyzed using flow cytometry. **f**, HCT116 cells were treated with 2 μM QW24 and the cells were imaged at the indicated time. Scale bars, 50 μm. **g**, HCT116, HT29 and CT26 cells were resuspended with 0, 1, 2, 4 μM QW24 in serum-free medium and seeded in transwell chambers for 12 h as descripted in **Methods** and the migrated cells were imaged and counted. Scale bars, 100 μm. Data are presented as mean ± s.d. (*n* = 3); **, *P* < 0.01; ***, *P* < 0.001

It has been reported that self-renewal could be the novel target for colorectal cancer therapy [18]. Sphere-formation assay, which recognizes and cultures stem cells, evaluates the abilities of self-renewal and differentiation for CICs in vitro on single cellular level [32]. Limiting dilution analysis (LDA) [33], which can calculate the frequency of CICs among cancer cells, is crucial for studying CICs. HCT116, the colorectal cancer stem-like cell line, was used in this study. As shown in Fig. 4a-d, QW24 suppressed the sphere-initiating cell frequency of HCT116 significantly, indicating QW24 inhibited the self-renewal ability of CICs. When cells were treated with the concentrations of 2 μM and 4 μM, the frequency of CICs decreased from 1/5 to 1/26 and 1/149 respectively (Fig. 4d). In addition, both the numbers of spheres in each well and the diameter of sphere decreased in dose-dependent manner with the treatment of QW24 (Fig. 4a, b and c). To further confirm the effect of QW24 on CICs, we used the colorectal CSCs makers CD133 and CD44 by the method of fluorescence-activated cell sorting (FACS). We found that with the treatment of QW24, the number of CICs decreased dose-dependently (Fig. 4e). Since death from colorectal cancer frequently results from tumor metastases [57] and at least 50% of patients develop metastases [58], we next explored the effect of QW24 on cancer cell migration in vitro. We firstly found that the QW24 treated cells had an obvious change of cellular morphology after 8-h treatment (Fig. 4f). Then we evaluated the effect of QW24 on colorectal cancer cells migration by transwell assay, and the result showed that QW24 inhibited the migration of colorectal cancer cells dramatically (Fig. 4g). These results together confirm that QW24 suppresses the self-renewal of colorectal CICs and cancer cell migration in vitro. Meanwhile, we determined the effect of QW24 on the proliferation of colorectal cancer cells with the same treatment condition as in transwell migration assay. HCT116, HT29 and CT26 cells were treated with QW24 at the concentrations of 0, 1, 2, 4 μM for 12 h, and the results showed that QW24 has no significant effect on cell viability at the same treatment condition as in transwell assay (Additional file 4: Figure S4A), which suggested that the reason that resulted in less cells translocating from the upper well to the down well was not due to the effect of QW24 on cell proliferation.

### QW24 inhibits the growth of colorectal tumor in subcutaneous xenograft model and down-regulates BMI-1 in vivo

**Fig. 5:**
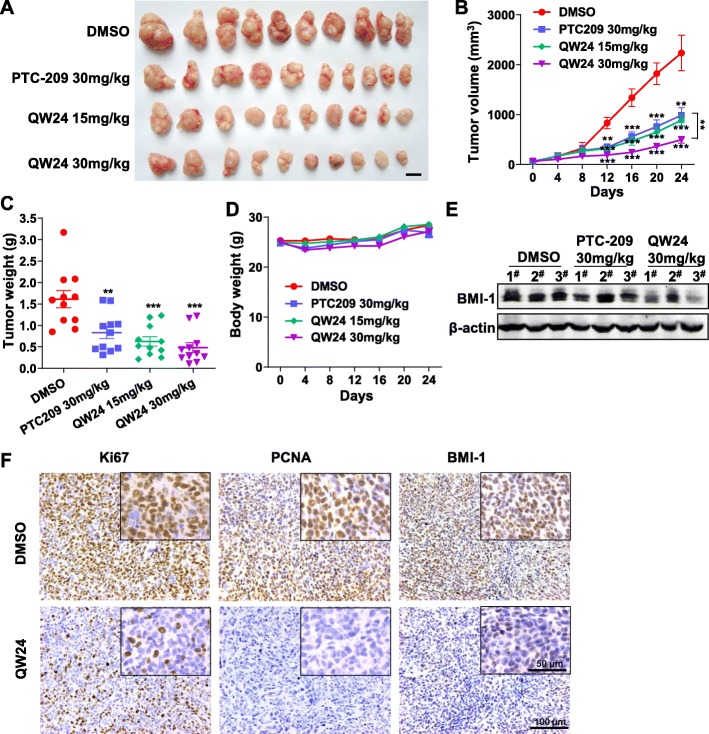
QW24 suppressed the colorectal tumor growth in subcutaneous xenograft model and down-regulated BMI-1 in vivo. **a-d**, HCT116 cells were injected into the right flank of BALB/c-nude mice. When the volumes of tumor nodules reached 100 mm^3^, the mice were randomly assigned to indicated groups and respectively i.p. injected with DMSO (*n* = 10), PTC-209 30 mg/kg (*n* = 10), QW24 15 mg/kg (*n* = 10) and 30 mg/kg (*n* = 10). After 24 days treatment, mice were sacrificed and tumors were harvested. Tumors image was photographed (**a**) and tumors were weighed (**c**). Tumor volume (**b**) and the mice body weight (**d**) were measured every 4 days. Scale bar, 1 cm. Data are presented as mean ± s.d. (*n* = 10); **, *P* < 0.01; ***, *P* < 0.001. **e**, Three representative tumor samples per group as indicated were lysed. BMI-1 protein level was measured by western blotting analysis. **f**, Images of xenografts treated with DMSO (control) and 30 mg/kg QW24 with corresponding IHC for Ki67, PCNA and BMI-1 were shown. The cropped region from each image is to enable a closer view. Scale bars, 100 μm or 50 μm as indicated

The above results demonstrated that QW24 had significant effects on inhibiting colorectal cancer cell proliferation, migration and self-renewal of colorectal CICs in vitro. To evaluate the therapeutic effect of QW24 on colorectal cancer in vivo, we performed the subcutaneous xenograft model in mice. The cancer stem-like cell line HCT116 was implanted subcutaneously to male BALB/c-nude mouse as described in **Methods**. As shown in Fig. 5a, b and c, both QW24 and PTC-209 inhibited tumor growth significantly. Notably, QW24 had a more significant tumor-inhibitory effect than PTC-209 at the same dose of 30 mg/kg/day. No obvious toxicity was noted in the animals during therapy experiments as assessed by mean body weight (Fig. 5d). Moreover, there was no obvious organ damage based on H&E staining results (Additional file 5: Figure S5A), and the mice had no abnormal behavior or side effect during treatment period. In addition, the tumor tissues were analyzed by western blotting and immunohistochemistry (IHC). Compared with the blank control group, the expression of BMI-1 in tumors was reduced in QW24 treated group (Fig. 5e). The proliferation maker Ki67 and PCNA decreased in QW24 treated group (Fig. 5f). These results confirm the inhibitory effects of QW24 on BMI-1 expression and tumor growth in vivo, and indicate anti-BMI-1 strategy could be a potential clinical therapy for colorectal cancer.

### QW24 inhibits the hepatic metastases of colorectal cancer

**Fig. 6:**
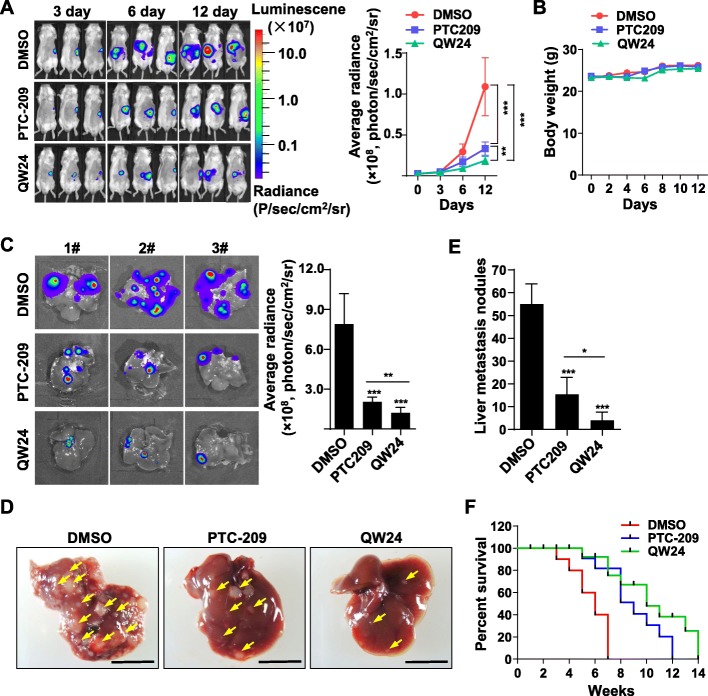
QW24 inhibited the hepatic metastases of colorectal tumor in vivo and extended the lifetime of mice. **a**, Male BALB/c-nude mice were anesthetized. CT26-luciferase colon cancer cells were surgically injected into the spleen. One week after injection, mice were administrated with splenectomy and mice were respectively i.p. injected with DMSO (*n* = 9), PTC-209 30 mg/kg (*n* = 9), QW24 30 mg/kg (*n* = 10) every day for 12 days. Hepatic metastases were monitored every 3 days using the IVIS Imaging System. Representative images of 3 mice per group were illustrated. Measurements of bioluminescent signals were acquired and analyzed using Living Image and Xenogen software (*n* ≥ 9). **b**, Mice body weight was measured every 2 days within treatment. **c-e**. After 12 days, mice were sacrificed and the livers of the mice were harvested, imaged to acquire bioluminescent signal and photographed to determine tumor cell dissemination. Representative images of hepatic metastases were showed, and the metastases were quantified and counted. Data are presented as mean ± s.d. (n ≥ 9); *, *P* < 0.05; **, *P* < 0.01; ***, *P* < 0.001. **f**, Animal model was generated as descripted in (**a**) and the mice were treated with DMSO (*n* = 10), PTC-209 30 mg/kg (*n* = 10) and QW24 30 mg/kg (*n* = 10) every day for 21 days. Then the survival rate of mice was recorded to 14 weeks and Kaplan-Meier survival curves were analyzed

As mentioned above, the tumor metastasis is the major reason for cancer lethal. The liver is the most common site of metastasis in colorectal cancer patients because of its anatomical situation regarding its portal circulation and the incidence of liver metastasis in colorectal cancer patients is about 35% [59]. Then we would explore the effect of QW24 on colorectal cancer metastasis in vivo and the liver metastasis model was performed as described in **Methods**. The results showed that QW24 administration suppressed the hepatic metastases effectively, and had a preferable effect than PTC-209 in vivo (Fig. 6a, c, d and e). The number of the tumor nodules decreased dramatically within QW24 treated mice (Fig. 6c and d). No obvious toxicity was observed with the measurement of mice body weight (Fig. 6b). Moreover, we used the same liver metastases model with new mice to determine the survival rate after the i.p. injection treatment with DMSO vehicle, PTC-209, and QW24 daily for 21 days and the survival of the mice were recorded to 14 weeks. The survival of mice was significantly prolonged with QW24 treatment, compared with vehicle control and PTC-209 administration group (Fig. 6f). These results established that QW24 prevents the hepatic metastases of colorectal cancer and improves the mice survival effectively.

## Discussion

Increasing studies have proved BMI-1 as an important therapeutic target in different cancers including colorectal cancer [17, 18, 29, 60–62]. BMI-1 plays roles in maintaining the viability and proliferation of colorectal cells and regulating CICs self-renewal [17]. The self-renewal property conferred by BMI-1 contributes to the maintenance of CICs in several different malignancies [18, 63–65] and brings about the chemotherapy resistance [66]. It has been proved that colorectal cancer cells are highly dependent on BMI-1, which works as the regulator maintaining the viability and proliferation of colorectal cancer cells and the self-renewal of CICs [18]. As reported [67], the lower BMI-1 expression predicted the longer survival of colorectal cancer patients. The finding that BMI-1 plays a significant role in colorectal CIC self-renewal opens the way to target CICs stemness with small-molecule inhibitor [68]. Here we report that the novel synthetic small molecule compound QW24 depletes BMI-1 at the protein level, significantly impacting the growth of CICs and reducing colorectal tumor growth and metastasis. Interestingly, we found that QW24 decreased BMI-1 protein level by protein degradation through autophagy-lysosome pathway and QW24 treatment induced autophagy in cells (Fig. 3). Notably, QW24 downregulated BMI-1 significantly with 2 μM treatment for 2 hours (Fig. 2b), at which time there was no obvious occurrence of autophagy except from the basal autophagy in cells (Fig. 3d, e and f). These results indicated that QW24 treatment induced BMI-1 protein degradation before the autophagy phenomenon. Recent studies reported that genetic or pharmacological inhibition of BMI-1 induced autophagy in cancer cells [69, 70]. Collectively, these results suggest QW24 depletes BMI-1 protein level and thereby induces autophagy.

While targeting BMI-1 has been reported the effective way to decrease tumor growth, the mechanisms of inhibiting BMI-1 differ in different system with context-dependence and are somewhat unclear. The BMI-1 inhibitor PTC-028 in ovarian cancer induced the hyperphosphorylation of BMI-1 and impacted BMI-1 function [29]. The small molecule PTC-209 downregulated BMI-1 by reducing mRNA level [18]. In our study, QW24 decreased BMI-1 protein level without obvious effect on BMI-1 mRNA level. Of note, treatment with QW24 induced the BMI-1 protein degradation, with the appearance of lower mobility bands (Fig. 1f and g), which resembled the effect of PTC-028 as reported [29]. Therefore, our results suggest QW24 treatment might induce the hyperphosphorylation of BMI-1 and then induce BMI-1 protein degradation. Mechanistically, we speculate that QW24 causes the hyperphosphorylation of BMI-1 that mediate the depletion of cellular BMI-1, coupled with the occurrence of autophagy that causes the degradation of hyperphosphorylated BMI-1 by lysosome. Our speculation is consistent with the results that the hyperphosphorylated band of BMI-1 was significantly rescued by lysosome inhibitor Chloroquine (Fig. 3a). Further research could confirm our speculation if the lower mobility hyperphospho bands disappeared and the basal BMI-1 level accumulated with phosphatase treatment. It has been reported that another BMI-1 inhibitor PTC596, which is efficacious in xenograft tumor models of glioblastoma, fibrosarcoma and leukemia, induces CDK1/2 binding to BMI-1 and CDK1/2-mediated phosphorylation of BMI-1 at N-terminal sites, leading to degradation of BMI-1 [28]. Thus, we speculate the mechanism that QW24 activate kinases and induces the hyperphosphorylation of BMI-1, then the non-functional phosphorylated BMI-1 protein would be up-taken by lysosome for degradation. Leading to the reduction of polycomb repressive complex 1 (PRC1) activity. The damaged BMI-1 proteins are recognized and degraded within cells, thereby eliminating the consequences of mistakes made by overexpressed BMI-1, resulting in the depletion of the CICs fraction and function. Further studies are needed to elucidate the mechanism of QW24 inhibiting colorectal cancer. Although others [18, 29] and our study identified several BMI-1 inhibitors, the direct target of these small molecules are still to be determined.

The inhibition of colorectal cancer cell growth by QW24 was reversed by BMI-1 overexpression with lentivirus infection (Fig. 1h), which suggests that colorectal cancer cells are dependent on BMI-1 for maintaining growth. A question needs to be considered is how QW24 affects normal human intestinal stem cells. BMI-1 is expressed in intestinal stem cells and contribute to the self-renewal of intestinal stem cells [71]. However, there was no obvious change in digestive function and body weight in QW24 treated mice (Fig. 5d and Fig. 6b), indicating that dose we used for significantly reducing tumor growth does not affect intestinal system obviously. The further oral administration could be performed to assess the effect of QW24 on animal intestinal system.

Targeting CICs self-renewal has been proposed as a therapeutic goal [72–74]. Strong evidences have proved that the key self-renewal regulator BMI-1 could be a central target for cancer therapy [15, 18]. A number of microRNAs and small-molecule compounds targeting BMI-1 regulating stemness have been identified [18, 20, 23, 27–29]. Future studied should be undertaken to transfer the preclinical research to clinical treatment. Our study provides the preclinical evaluation of the effect of targeting BMI-1 on cancer treatment in vitro and in vivo. Further studies are needed to transform our preclinical research into clinical cancer therapy. ADME/Tox drug properties, absorption, distribution, metabolism, elimination and toxicity, are properties crucial to the final success of a drug candidate [75]. Since QW24 was therapeutically potent against colorectal cancer in animal models, drug-like properties of QW24 are needed to be evaluated. Oral delivery is the most desirable route of drug administration [75]. Therefore, it is important to determine the antitumor activity of QW24 by oral administration in animal model. The pharmacokinetics-pharmacodynamic model should be performed to determine the dose level and drug exposures necessary for QW24 to achieve potent antitumor activity in vivo. The preclinical pharmacokinetics of QW24 should be evaluated to determine the properties of absorption, distribution, elimination, and bioavailability. A drug that is absorbed orally is transported via the portal circulation to the liver, where it is subjected to hepatic metabolism followed by bile or via the kidneys. The key enzymes for drug metabolism are the isoforms of the cytochrome P450 (CYP) family [75]. Thus, the effect of QW24 on the activity of CYP enzymes should be evaluated. These pharmacokinetics parameters would provide the basis for QW24 in future clinical trials. Moreover, drug toxicity is a crucial drug property. For a desirable drug, the plasma level required to exert a toxic effect would be significantly higher than that required for therapeutic efficacy [75]. In our study, the dose of QW24 for colorectal cancer therapy in mice model did not exert obvious toxicity to mice, based on the results that there was no obvious body weight decrease, organ damage, abnormal behavior or side effect during treatment. More details about QW24 toxicity could be assessed further.

## Conclusion

In summary, the novel small molecule QW24 downregulated BMI-1 by inducing the protein degradation through autophagy-lysosome pathway. Notably, QW24 depletes self-renewal of CICs and migration of cancer cells significantly. Moreover, QW24 reduces colorectal tumor growth, inhibits the tumor metastasis effectively and increases the mice lifetime in our preclinical study. Therefore, QW24 could potentially be used as an effective therapeutic agent for colorectal cancer treatment.

## Additional files

Additional file 1: **Figure S1.** The screening process of compounds to down-regulate BMI-1. **A,** Schematic illustrates the screening process of QW24 which can down-regulate BMI-1. **B** and **C,** The compounds with IC50 less than 1 μM in HCT116 and HT29 cells were listed (**B**) and the chemical structures of the compounds were shown (**C**). **D,** HCT116 cells were treated with the indicated compounds and concentrations for 12 h. Cells were lysed and BMI-1 protein level was measured by western blotting analysis. **E,** HCT116 cells were treated with the indicated concentrations of QW24 or QW30 for 24 h. Cells were lysed and BMI-1 protein level was measured by western blotting analysis. **F,** The normal cell lines, including human normal liver cell L02, human skin fibroblast cell HAF, human normal colon epithelium cell NCM460 and human umbilical vein endothelial cell HUVEC, were seeded in 96-well plates (3000 cells/well) and treated with 0, 0.5, 1, 2, 4 μM of QW24 after cells were attached. After 72 h incubation, cell growth was measured by SRB assay. Data are presented as mean ± s.d. (*n* = 5); **, *P* < 0.01; ***, *P* < 0.001. (DOCX 100 kb)

Additional file 2: **Figure S2.** QW24 inhibits colorectal cancer cells proliferation more significantly than PTC-209. **A,** HCT116, HT29 and HCT8 cells were treated with indicated concentrations of PTC-209 or QW24 for 7 days, and the cell colonies were counted. Data are presented as mean ± s.d. (*n* = 3); *, *P* < 0.05; **, *P* < 0.01; ***, *P* < 0.001. **B,** HT29, HCT8 and CT26 cells were seeded in 96-well plates (3000 cells/well) and treated with 0, 0.5, 1, 2, 4 μM of QW24 or PTC-209 after cells were attached. After 72 h incubation, cell growth was determined by SRB assay. Data are presented as mean ± s.d. (*n* = 5); *, *P* < 0.05; **, *P* < 0.01; ***, *P* < 0.001. (DOCX 210 kb)

Additional file 3: **Figure S3.** BMI-1 protein level is higher in cancer cells than normal cells and overexpression of BMI-1 correlates with poor patient survival in colorectal cancer. **A,** BMI-1 protein levels in various cells were measured by western blotting analysis, including human breast cancer cells MDA-MB-231, lung cancer cells A549, ovarian cancer cells ES2, liver cancer cells HepG2, prostate cancer cells PC3 and DU145, colorectal cancer cells HT29 and HCT116, as well as human normal liver cell L02, human skin fibroblast cell HAF, human normal colon epithelium cell NCM460 and human umbilical vein endothelial cell HUVEC. **B,** BMI-1 is differently expressed in colorectal cancer and normal tissues, as indicated by UALCAN (http://ualcan.path.uab.edu) [76]. **C,** Higher expression levels of BMI-1 showed poor survival rates in colorectal cancer patients, as indicated by The Human Protein Atlas (https://www.proteinatlas.org) [77]. (DOCX 82 kb)

Additional file 4: **Figure S4.** **A,** HCT116, HT29 and CT26 cells were seeded in 96-well plates and treated with 0, 1, 2, 4 μM of QW24 after cells were attached. After 12 h incubation, cell growth was determined by SRB assay. Data are presented as mean ± s.d. (*n* = 5); n.s., Not statistically significant. (DOCX 40 kb)

Additional file 5: **Figure S5.** The H&E staining of mice organs in subcutaneous tumor xenografts animal model. **A,** In subcutaneous tumor xenografts animal model, after mice were sacrificed, the hearts, livers, spleens, lungs and kidneys from DMSO and QW24 (30 mg/kg) treated group were harvested for H&E staining and imaged. Scale bars, 100 μm. (DOCX 196 kb)

## Data Availability

All data generated or analyzed during this study are included in this article and its supplementary files.

## Abbreviations

BSA: Bovine serum albumin
CIC: Cancer-initiating cell
CRC: Colorectal cancer
CSC: Cancer stem cells
DMSO: Dimethyl sulfoxide
EGF: Epidermal growth factor
FGF: Fibroblast growth factor
H&E: Hematoxylin-eosin
i.p: Intraperitoneally
IC50: Half maximal inhibitory concentration
IHC: Immunohistochemistry
LDA: Limiting dilution analysis
PRC1: Polycomb repressive complex 1
s.d: Standard deviation
SFM: Serum-free medium
SRB: Sulforhodamine B
