# Supplementary material for: A novel BMI-1 inhibitor QW24 for the treatment of stem-like colorectal cancer
Source: J Exp Clin Cancer Res. 2019 Oct 22;38:422. doi: 10.1186/s13046-019-1392-8 (PMC6805542; doi:10.1186/s13046-019-1392-8)

## Supplementary figure S1

A

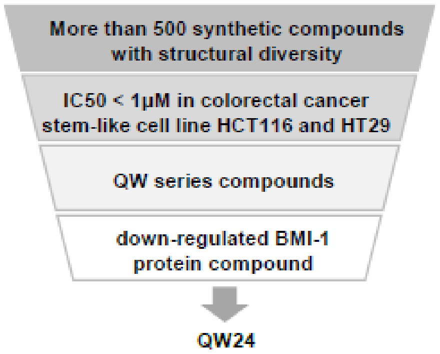


B

| Compounds | IC50 in colorectal cancer cell lines (μM) | |
|---|---|---|
| | HCT116 | HT29 |
| QW07 | 0.50 | 0.51 |
| QW30 | 0.56 | 0.59 |
| QW10 | 0.57 | 0.62 |
| QW24 | 0.69 | 0.86 |
| PTC-209 | 1.67 | 1.36 |

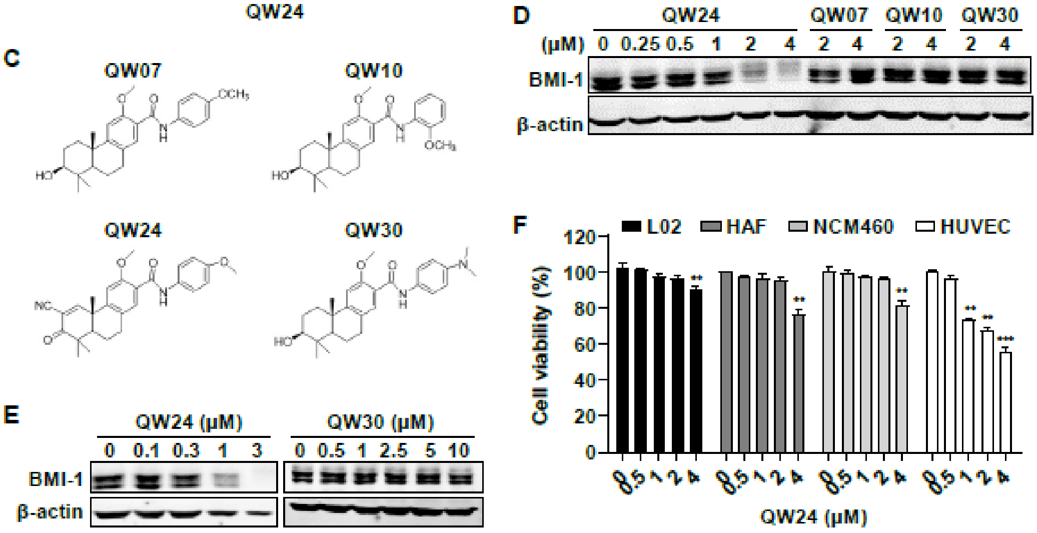

Supplement: Supplementary file 1 [file 13046_2019_1392_MOESM1_ESM.docx]
