# Supplementary material for: A novel BMI-1 inhibitor QW24 for the treatment of stem-like colorectal cancer
Source: J Exp Clin Cancer Res. 2019 Oct 22;38:422. doi: 10.1186/s13046-019-1392-8 (PMC6805542; doi:10.1186/s13046-019-1392-8)

Supplementary figure S3

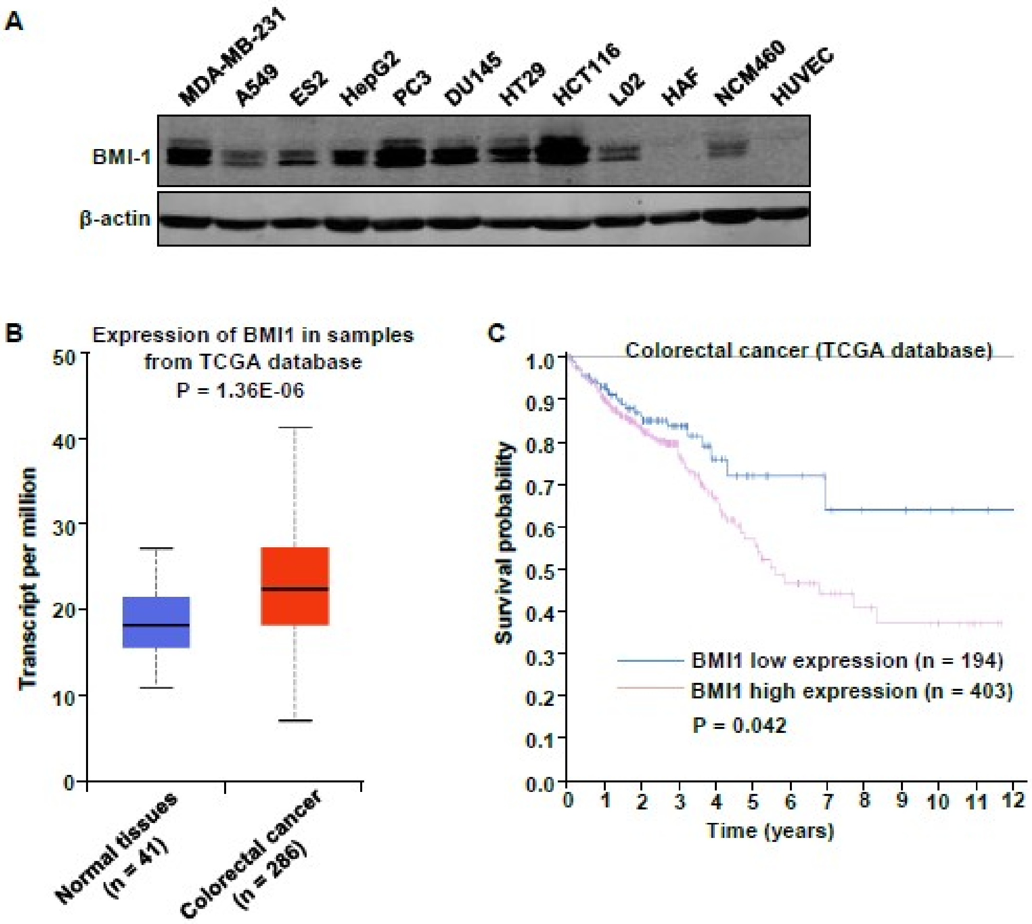

Supplement: Supplementary file 3 [file 13046_2019_1392_MOESM3_ESM.docx]
